# Development and Validation of Polypharmacy-Related Psychological Distress Scale (PPDS): A Preliminary Study

**DOI:** 10.3390/bs15050707

**Published:** 2025-05-21

**Authors:** Cheng Cheng, Xiao Chen, Junqiao Wang, Martin Christensen

**Affiliations:** 1 School of Nursing, Fudan University, Shanghai 200030, China; 2 School of Nursing, Hong Kong Polytechnic University, Hung Hom, Kowloon, Hong Kong SAR, China; 3 Nursing Key Laboratory of Sichuan Province, Chengdu 610041, China

**Keywords:** factor analysis, outcome assessment, psychometrics, polypharmacy, psychological distress

## Abstract

Polypharmacy is an increasing concern in healthcare due to its potential to cause adverse drug reactions and medication non-adherence. The evidence has identified a connection between psychological distress and polypharmacy, yet there is a lack of validated instruments to measure this specific type of distress. This study aims to develop and validate the Polypharmacy-related Psychological Distress Scale. This study followed a rigorous scale development procedure, encompassing item creation, scale development, and scale evaluation. A multi-method design incorporated a literature review, the Delphi method with eight experts, and a cross-sectional survey with 97 participants. A comprehensive range of psychometric tests, including content validity, face validity, concurrent validity, internal consistency, and construct validity, were utilized to assess the goodness of the instrument—the finalized scale comprised four items. Content validity results were deemed satisfactory based on CVI for item (I-CVI) and CVI for scale (S-CVI). Face validity was established through the incorporation of participant feedback. A significant correlation was found between the Patient Health Questionnaire-4 and the Polypharmacy-related Psychological Distress Scale, with a correlation coefficient of 0.444 (*p* < 0.001). The scale demonstrated acceptable reliability, with a Cronbach’s alpha of 0.790 and a McDonald’s omega of 0.937. A confirmatory factor analysis revealed a unidimensional structure. To summarize, the Polypharmacy-related Psychological Distress Scale showed satisfactory reliability and validity in this preliminary study. It holds promise for use by healthcare professionals to assess psychological distress in the target population, pending further validation.

## 1. Introduction

Polypharmacy, the concurrent use of at least five medications by an individual, has become increasingly common in clinical practice (Masnoon et al., 2017). The prevalence of polypharmacy has been rising due to various factors, such as an ageing population, the increasing burden of chronic diseases, and the availability of a wide range of medications (Delara et al., 2022). According to data from the National Health and Nutrition Examination Survey (NHANES), the prevalence of polypharmacy among adults in the USA has shown a steady upward trend from 1999–2000 to 2017–2018, with the overall percentage increasing from 8.2% to 17.1%. This trend was even more evident among older populations, with a significant rise from 23.5% to 44.1% (Wang et al., 2023). A systematic review revealed that, despite variations in operational definitions across studies, the polypharmacy prevalence varied from 2.6% to 86.6% (Nicholson et al., 2024).

While polypharmacy can be therapeutically beneficial, it usually poses significant risks, such as adverse drug reactions, drug interactions, therapeutic failure, and increased healthcare costs (Mair et al., 2020). In addition to these physical health concerns, research has linked polypharmacy to an individual’s psychological well-being (Palapinyo et al., 2021). For example, researchers have found that older adults who are prescribed multiple medications are at a higher risk of experiencing anxiety, depression, and a reduced quality of life (Cheng & Bai, 2022). The relationship between psychological distress and polypharmacy is complex and bidirectional, influenced by a variety of factors. Side effects and drug interactions from multiple medications can exacerbate psychological distress (Celano et al., 2011), while the burden of experiencing numerous prescriptions adds to stress (Awad et al., 2020). Conversely, individuals with psychological distress often have multiple health conditions requiring various medications, leading to polypharmacy. Fragmented healthcare systems and the lack of comprehensive medication reviews further complicate this issue (Ando et al., 2023). Socioeconomic factors, such as lower income and older age, increase the likelihood of both polypharmacy and psychological distress (Kim et al., 2024). In a qualitative study (Smith et al., 2019), older adults experiencing polypharmacy reported numerous challenges with their medication regimens. They expressed a desire to reduce their medication burden to improve their daily well-being. Addressing this intricate relationship necessitates a holistic approach that emphasizes coordinated care, regular medication reviews, and robust mental health support.

Despite the growing recognition of the psychological burden associated with polypharmacy, there remains a notable gap in standardized tools designed specifically to assess the emotional and cognitive impact of managing multiple medications. Existing instruments, such as those assessing medication-related burden or adherence, tend to emphasize functional aspects, including the number of prescribed medications, regimen complexity, and patient compliance (Iqbal et al., 2022). While such measures are crucial for identifying practical barriers to medication use, they often overlook the emotional distress, anxiety, and perceived loss of autonomy that many individuals experience as a result of complex and prolonged pharmacotherapy. This psychological dimension is particularly relevant for older adults, who are disproportionately affected by multimorbidity and are more likely to be prescribed multiple medications over extended periods.

### Theoretical Framework

The Polypharmacy-related Psychological Distress Scale (PPDS) was designed to quantify and operationalize the psychological distress that arises from polypharmacy. This concept is firmly rooted within the framework of the biopsychosocial model of health, which acknowledges the interplay of biological, psychological, and social determinants in health and disease (Engel, 1980). More specifically, the PPDS is intended to capture the spectrum of emotional and cognitive reactions that individuals may encounter while experiencing multiple medications. It might help us to better understand the psychological impact of polypharmacy and to identify individuals who may be at risk of experiencing distress. Such an instrument can also be used to evaluate the effectiveness of interventions aimed at reducing polypharmacy-related psychological distress and improving the overall well-being of individuals with complex medication regimens.

As global healthcare systems are facing significant challenges in managing patients who are prescribed multiple medications, the need for standardized tools to measure polypharmacy-related psychological distress has become pressing (Ágh et al., 2021; Khezrian et al., 2020). The PPDS was developed to measure this distress, though it may not fully capture its complexity, highlighting the need for the PPDS.

This study aims to develop and validate a scale for assessing psychological distress related to polypharmacy.

## 2. Materials and Methods

**Figure 1:**
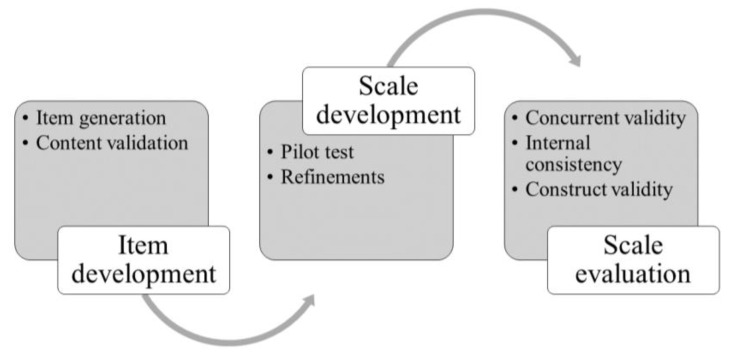
Process of scale development.

In this study, we adhered to the COSMIN reporting guidelines for studies on measurement properties (Gagnier et al., 2021) and followed the general procedure of scale development, which included three major phases: (1) item development, (2) scale development, and (3) scale evaluation (Boateng et al., 2018). See Figure 1.

### 2.1. Phase 1: Item Development

#### 2.1.1. Item Generation

The first step in developing the scale involved generating a comprehensive pool of potential items to capture the emotional dimensions of the PPDS. This process utilized two primary approaches. First, we conducted pilot qualitative interviews with nine individuals who were experiencing polypharmacy. The inclusion and exclusion criteria were the same as those used in the cross-validation phase and are described below. The sample consisted of 9 participants (5 females and 4 males), aged between 62 and 71 years, with a mean age of 66.4 years. The most reported chronic conditions were hypertension, diabetes, and heart disease, with four participants reporting multimorbidity.

These interviews aimed to explore their lived experiences, focusing on emotional and psychological challenges associated with taking multiple medications. The participants were asked open-ended questions about their feelings, concerns, and coping strategies related to polypharmacy. The interviews were transcribed and thematically analyzed to identify recurring emotional patterns. Five items regarding worries, burden, quality of life, adherence, and distress in the context of the daily experience of polypharmacy were developed by the first two authors based on the interviews and were subsequently reviewed by the third author.

Second, we retrieved relevant information from two systematic reviews of the existing literature on polypharmacy (Cheng et al., 2023; Eriksen et al., 2020). These reviews offered a broader perspective by identifying the established challenges and emotional stressors experienced by individuals experiencing polypharmacy. Two additional items regarding anxiety and frustrations concerning polypharmacy were identified by the first two authors. To complement this literature-based approach, we also sought insights from a panel of six experts in nursing, geriatrics, and clinical pharmacy. Their input ensured that the generated items reflected diverse professional viewpoints and were anchored in clinical significance. Those items were designed to reflect the experiences and concerns of individuals with polypharmacy, such as worries about potential drug interactions and feelings of being overwhelmed from experiencing medication regimens. Three items were combined, leaving four items remaining for further validation. The original language for these items was Chinese.

#### 2.1.2. Content Validation

The next step involved conducting a content validation study to assess the relevance and comprehensiveness of the items. We employed a Delphi technique consisting of two survey rounds (McKenna, 1994). A panel of eight experts in nursing, geriatrics, public health, and clinical pharmacy reviewed the items and provided feedback on their clarity, relevance, and comprehensiveness (Polit et al., 2007). The selection criteria of the experts included the following: (1) being engaged in clinical or research work related to chronic disease care or health management for more than 10 years; (2) holding senior professional titles; (3) being willing to participate in this study. Those who refused to participate in this study were excluded. The content validation panel was composed of experts who were different from those involved in providing the initial input during the item development phase.

We collected data through electronic consultation questionnaires via email. We used the content validity index (CVI) approach for content validity in instrument development, which was computed using the Item-CVI (I-CVI) and the Scale-CVI (S-CVI) (Polit & Beck, 2006). Experts could also clarify their opinions and suggestions in the blank space provided. Based on the expert feedback, the initial pool of items was refined to ensure the scale effectively captured the multidimensional nature of the PPDS. Several items were initially too broad and were revised to target specific aspects of polypharmacy-related distress. For example, the original item, “Have you felt worried because of the names and usages of medications?” was modified to “Have you felt worried or nervous in the past two weeks because of the names and usages of different medications?” to enhance clarity and ensure a clear timeframe for responses. Additionally, the introduction to the scale was refined to provide clearer instructions for respondents.

### 2.2. Phase 2: Scale Development

The refined pool of items was then pilot-tested with a sample of 15 people with polypharmacy to assess the feasibility and acceptability of the scale. The inclusion and exclusion criteria were the same as those used in the cross-validation phase and are described below. The pilot testing involved administering the scale to a group of ten participants and collecting feedback on the clarity, relevance, and comprehensiveness of the items. Based on the feedback from the pilot testing, further refinements, such as the introduction of the PPDS, were made to the scale to ensure that it was clear, relevant, and acceptable to individuals with polypharmacy.

### 2.3. Phase 3: Scale Assessment

#### 2.3.1. Study Design

The final phase was a psychometric evaluation of the instrument to assess its reliability and validity. The current study employed a cross-sectional design, targeted to a group of people with polypharmacy. This study was conducted between November 2023 and July 2024.

#### 2.3.2. Sample

The participants were conveniently recruited from a community healthcare center in north Anhui, China. The inclusion criteria for the participants included the following: (1) aged 18 or over; (2) had at least one chronic disease, such as cancer, hypertension, type 2 diabetes, heart disease, stroke, chronic kidney disease, chronic obstructive pulmonary disease (COPD), musculoskeletal disorders, cervical and lumbar diseases, and digestive system disorder; (3) regularly took more than five or more drug prescriptions. The exclusion criteria included the following: (1) not able to understand the questions, and (2) refusal or withdrawal from participation. Individuals with self-reported histories of mental disorders were excluded from participation. Additionally, the participants who were hospitalized or recruited from hospital settings during the study period were excluded to minimize the risk of overestimating the psychological effects of medications. Those who declined to participate were also excluded from this study.

Based on the rough rating scale for factor analysis, which suggests that a sample size of at least 100 is a reasonable absolute minimum for confirmatory factor analysis (CFA) (Kline, 1994), we purposively distributed 120 paper-based questionnaires, anticipating a 15% non-response rate.

#### 2.3.3. Measures

Sociodemographic characteristics, including age, sex, living status, and education level, were collected. Clinical information, including the type of chronic disease and duration of chronic disease, was obtained.

The Polypharmacy-related Psychological Distress Scale (PPDS) is a short instrument designed to assess psychological distress related to polypharmacy. The original scale has four items for psychological distress about medications with a Likert reply: “1” = “Completely disagree”, “2” = “Disagree”, “3” = “Unsure”, “4” = “Agree”, and “5” = “Completely agree”. Participants were asked to mark their level of agreement or disagreement with a series of items. The scores were summed to obtain a total score ranging from 5 to 20, with higher scores indicating greater feelings of distress. Those items test different aspects of an individual’s experience with taking multiple medications over the past two weeks.

Table 1 reports the items of the PPDS. The first item, “Have you felt worried or nervous in the past two weeks due to taking a lot of medications every day?”, tests the emotional burden of polypharmacy, specifically the frustration or annoyance associated with experiencing multiple daily medications. The second item, “Have you felt worried or nervous in the past two weeks because of the names and usages of different medications?”, examines the impact of the medication regimen complexity on the individual, including confusion or difficulty with understanding the medication names and instructions. The third item, “Have you felt worried or nervous in the past two weeks about taking medications on time and in the correct dosage?”, assesses the person’s experience with medication adherence, including their level of satisfaction or frustration with following the prescribed schedule and dosage. The fourth item, “Have you felt worried or nervous in the past two weeks about the necessary precautions when taking medications?”, measures the inconvenience caused by special instructions or precautions required for medication use.

The Patient Health Questionnaire-4 (PHQ-4) is a short instrument designed to assess depression and anxiety disorders, comprising the initial two items from both the PHQ-9 and GAD-7 measures (Kroenke et al., 2009). It is a widely used and well-validated measure of psychological distress in terms of depression and anxiety (Wicke et al., 2022). Each item is rated on a 4-point Likert scale, ranging from 0 (not at all) to 3 (nearly every day). The overall PHQ-4 scores can vary between 0 and 12. Higher scores indicate more depression and anxiety symptoms. In this study, the Cronbach’s alpha of the PHQ-4 was 0.936.

#### 2.3.4. Data Collection

Two research assistants were trained and recruited by the primary investigator to identify potential participants. A healthcare professional at the research site helped with participant recruitment using telephone methods and flyer advertisements. Once a potential participant was identified, they were asked if they were willing to participate in the study. People who agreed to participate would be asked to sign consent forms and were enrolled in the study. To ensure the privacy of all participants, the questionnaires were kept anonymous. Before distributing the questionnaires, a comprehensive briefing on the aim of the study and the instructions for completing the scale were provided. The survey was self-reported and paper-based, with research assistants available to assist the participants as needed. The primary investigator administered the whole process.

#### 2.3.5. Data Analysis

Descriptive statistics were used to analyze the sociodemographic characteristics. The normality of item responses was assessed using skewness and kurtosis. Concurrent validity was examined by the correlation between the PPDS and the PHQ-4 (Lin & Yao, 2014). Internal consistency was examined by Cronbach’s alpha coefficient (Revicki, 2014) and McDonald’s omega (ω) (Trizano-Hermosilla & Alvarado, 2016), with values ≥ 0.70 considered acceptable. An item-total correlation test was employed to verify the consistency of the items and ensure their alignment with the overall scale.

Given that exploratory factor analysis (EFA) is inappropriate for a four-item scale, construct validity was assessed using CFA to test the unidimensionality of the PPDS. The CFA was performed using the diagonally weighted least squares (DWLS) estimator to account for the ordinal nature of the data. Model fit was evaluated using multiple fit indices: comparative fit index (CFI; acceptable ≥ 0.90, good ≥ 0.95), Tucker–Lewis index (TLI; acceptable ≥ 0.90, good ≥ 0.95), root mean square error of approximation (RMSEA; acceptable ≤ 0.08, good ≤ 0.05), and standardized root mean square residual (SRMR; acceptable ≤ 0.08, good ≤ 0.05) (Brown, 2015). Factor loadings were examined, with values ≥ 0.50 considered acceptable for convergent validity.

All analyses were computed by IBM SPSS Statistics for Windows, Version 25.0. Armonk, NY: IBM Corp. and JASP (Version 0.16.3).

## 3. Results

### 3.1. Content Validity

The panel with eight experts checked and approved all the included items. The I-CVI ranged from 0.82 to 0.93, and the S-CVI was 0.89, indicating good content validity of the items based on the recommended guidelines (Cicchetti & Sparrow, 1981).

### 3.2. Face Validity

The PPDS was administered to five participants, and four out of five suggested the item wording was clear and easy to understand. The statement of items 3–4 was slightly modified based on the participants’ feedback. For instance, we replaced the words “frustrated or overwhelmed” with “worried or nervous” to better capture the respondent’s emotional experience of polypharmacy. Also, we provided a statement regarding polypharmacy (e.g., an explanation of prescribed drugs) below the scale.

### 3.3. Cross-Sectional Validation Study

Table 2 reports the sociodemographic characteristics of the participants. A total of 97 participants completed and returned the questionnaire, resulting in a response rate of 80.8%. Out of the 97 participants who returned their questionnaires, 91 provided full data. The sample consisted of 54 males (40.7%) and 37 females (59.3%). The majority of participants were aged between 60 and 79 years (59.4%). The majority had received a fundamental level of education (69.2%) and reported having a stable income (70.3%). A significant proportion of participants resided with their families (78.0%). The most common chronic diseases were hypertension (74, 81.3%), diabetes (51, 56.0%), heart diseases (33, 36.2%), cerebrovascular diseases (30, 33.0%), and arthritis (21, 23.1%). The PPDS scores ranged from 4 to 18, with a mean of 11.9 ± 3.0. The 25th percentile was 10.0, the median (50th percentile) was 12.0, and the 75th percentile was 14.0.

Table 3 presents the distribution of responses across the four items of the PPDS.

### 3.4. Concurrent Validity

Given the normal distribution of the data, the Pearson correlation coefficient was used to calculate the correlation between the PPDS scores and the PHQ-4 scores. The correlation between the two instruments was moderate (r = 0.444, *p* < 0.001).

### 3.5. Internal Consistency

Table 4 reports the results of Internal consistency. The Cronbach’s alpha coefficient for items 1 to 4 was 0.790 and the McDonald’s omega was 0.937 (95% CI: 0.916–0.958). All coefficients were > 0.70, indicating acceptable-to-excellent reliability in terms of internal consistency (Nunnally & Bernstein, 1994).

### 3.6. Inter-Item Correlation

Table 5 reports the item-total correlation of the four items. The corrected inter-item correlation values were all positive and above 0.60 (*p* < 0.01), except for item 4, indicating a moderate-to-strong correlation (Gignac & Szodorai, 2016).

### 3.7. CFA Factor Analysis

A CFA was conducted to assess the construct validity of the PPDS. Given the theoretical framework and the limited number of items, a unidimensional model was specified. The model fit indices indicated good model fit: CFI = 0.986, TLI = 0.987, RMSEA = 0.06 (90% CI: 0.03–0.09), and SRMR = 0.04, supporting the unidimensional structure of the scale.

Factor loadings for the four items were 0.65, 0.69, 0.73, and 0.80, all exceeding the recommended threshold of 0.50, indicating strong item contributions to the latent construct.

## 4. Discussion

The studies detailed above were conducted to develop a measure to examine psychological distress concerning polypharmacy. Our primary objective was to develop a simple, scalable tool capable of examining polypharmacy-related psychological distress, adhering to best practice processes (Boateng et al., 2018). Our findings indicated that the PPDS holds potential for utility and scalability in identifying adverse emotional responses associated with polypharmacy. This tool might enable the application of straightforward interventions to assist individuals experiencing psychological distress due to polypharmacy, thereby potentially improving their overall well-being.

The expert panel’s review and approval of the items, along with the I-CVI values ranging from 0.82 to 0.93 and an S-CVI of 0.89, suggested that the items exhibit strong content validity. According to the Cicchetti and Sparrow (1981) guidelines, these results supported the appropriateness of the items in measuring the intended construct, reflecting the panel’s consensus on their relevance and clarity. The high I-CVI and S-CVI values further indicated that the scale is well-aligned with the target concept, providing a reliable foundation for subsequent stages of this study.

The moderate correlation between the PPDS and PHQ-4 scores (r = 0.444, *p* < 0.001) supported the concurrent validity of the PPDS, suggesting that the scale aligns with other established measures of psychological distress.

The Cronbach’s alpha of 0.790 and McDonald’s omega of 0.937 for the PPDS indicated that the scale demonstrates good internal consistency, as values above 0.70 are considered acceptable-to-excellent (McDonald, 1999; Nunnally & Bernstein, 1994). These findings reinforced the reliability of the PPDS as a consistent measure of polypharmacy-related psychological distress.

The positive and above 0.60 corrected inter-item correlation values for most items suggested that the items are well correlated with the overall scale, indicating a good level of internal consistency. However, the exception of Item 4, which did not show a strong correlation, may require further examination or revision. According to Gignac and Szodorai (2016), moderate-to-strong correlations among items suggested that the scale measures a cohesive construct, supporting the scale’s reliability.

Given the scale’s theoretical framework and its limited number of items, a unidimensional model was specified. The results of the CFA indicated good model fit, with the fit indices meeting or exceeding the recommended thresholds. The CFI = 0.986 and the TLI = 0.987 both indicated an excellent fit, suggesting that the model accurately represents the underlying structure of the PPDS. The RMSEA = 0.06, 90% CI: 0.03–0.09 was within an acceptable range, although the upper bound of the confidence interval approached the upper threshold for acceptable fit. The SRMR = 0.04 further supported the model, as it fell well below the recommended cutoff of 0.08.

In terms of factor loadings, the four items demonstrated satisfactory contributions to the latent construct, with values ranging from 0.65 to 0.80, all exceeding the recommended threshold of 0.50. These findings suggest that each item on the PPDS adequately reflects the underlying construct of the PPDS. Specifically, the item with the highest loading (0.80) appears to be the most representative of the latent factor, while the item with the lowest loading (0.65) still contributes significantly to the overall scale. The unidimensionality of the PPDS is further supported by these results, as the items appear to tap into a single underlying construct. This was consistent with the theoretical framework of the scale, which theorizes that psychological distress related to polypharmacy is best represented as a single, unified factor. The good fit indices and adequate factor loadings suggest that the PPDS is a valid instrument for measuring psychological distress in individuals experiencing polypharmacy.

Although Item 4 (“Have you felt worried or nervous in the past two weeks about the necessary precautions when taking medications?”) showed a lower item-total correlation and its removal would slightly increase the Cronbach’s alpha, we chose to retain it based on theoretical and practical considerations. Theoretically, psychological distress related to polypharmacy is a multidimensional construct. While Items 1–3 focus on volume, complexity, and adherence, Item 4 uniquely reflects anticipatory anxiety associated with the safety and self-management of medications, such as concerns about interactions, side effects, or dietary restrictions. These concerns are consistent with frameworks such as Leventhal’s common sense model (Leventhal et al., 2016) and the burden of treatment theory (May et al., 2014), which identify emotional and cognitive responses to treatment demands. Practically, precaution-related anxiety frequently emerges in clinical encounters, particularly among individuals managing multiple medications for chronic conditions. Retaining Item 4 enhances the content validity of the scale by ensuring it captures a broader range of real-world concerns, even if this comes at a minor cost to internal consistency. However, while the current findings are promising, future research may benefit from exploring the scale’s performance in larger and more diverse populations, as well as evaluating its predictive validity and sensitivity to changes over time. Furthermore, additional psychometric properties, such as test–retest reliability and criterion validity, should be assessed to strengthen the evidence for the scale’s use in clinical settings.

The PPDS’s design allows for the early identification of individuals at risk, facilitating timely and appropriate interventions. Recent research has emphasized a growing preference for shorter scales to measure psychological constructs, driven by the difficulties associated with longer scales. These included fatigue (Penson et al., 2020), anger (Kannis-Dymand et al., 2019), and fear (Ventura-León et al., 2020). Additionally, extended scales might skew the participants’ cognitive and emotional responses and require more assessment time and cost (Kemper et al., 2019). Therefore, scholars often favor shorter scales. Although there are short versions of psychological distress in terms of anxiety and depression measures, they do not examine emotional states generally concerning polypharmacy, and they are not as short as the PPDS. For example, the Kessler Psychological Distress Scale (Kessler et al., 2002) has six items and the short version of the Malaise Inventory has nine items (Ploubidis et al., 2019).

Notably, the current study’s sample consisted solely of older adults, which is a critical consideration when interpreting the results. The experience of psychological distress in older adults with polypharmacy may be influenced by age-related factors, such as cognitive decline, social isolation, and comorbidities, all of which can impact the manifestation and expression of emotional distress. In this study, the sample was primarily composed of older individuals who had lower educational attainment, with 100% of participants not having a post-secondary degree. This differs from international data, such as those conducted in Australia, where around 20% of older adults report having completed post-secondary education (Australian Bureau of Statistics, 2016). The disparity can be attributed to regional differences in educational access, as older adults in China, particularly in rural areas, have historically faced barriers to higher education. Therefore, the findings of this study should be interpreted with these demographic differences in mind, as education level and regional factors may influence both medication use and psychological distress. It is essential to consider the age-specific and socio-cultural context when evaluating the emotional distress of older adults with polypharmacy. Healthcare professionals must also consider the unique challenges and vulnerabilities associated with ageing—such as the impact of lower educational attainment—when assessing and addressing the psychological needs of this population.

### 4.1. Limitations

This study has several limitations that should be considered when interpreting the findings. First, the development of the PPDS was primarily based on classical measurement theory (CMT), which assumes unidimensionality. This assumption may not fully capture the complexity of psychological distress associated with polypharmacy, as distress is likely to be multidimensional, encompassing aspects such as medication-related anxiety, frustration, and practical challenges. The focus of CMT on group-level analyses may also overlook individual differences in distress, potentially leading to measurement error.

Second, the self-report nature of the PPDS introduces potential biases, including social desirability bias, recall bias, and limited self-awareness. These biases may affect the accuracy of the participants’ responses, leading to either overestimation or underestimation of distress levels. Additionally, while the PPDS is a brief and quick tool, its brevity may limit its ability to capture the full depth of polypharmacy-related psychological distress.

Third, the limited pilot testing conducted with a small group of participants may not have captured all potential issues with the PPDS. Also, while this sample size approaches the lower threshold for CFA, it may still limit the statistical power and generalizability of the findings. The relatively small sample size also restricts the complexity of the model that can be reliably tested, increases the likelihood of sampling error, and may affect the stability of factor loadings. Future studies with larger and more diverse samples are recommended to confirm the factor structure and enhance the robustness and external validity of the scale.

Fourth, consumer perspectives were not included in the scale development process. Although the PPDS is designed to assess the patients’ subjective psychological distress, the expert panel consisted solely of healthcare professionals and researchers. The absence of direct input from individuals experiencing polypharmacy may have limited the scale’s ability to fully capture the nuances of their experiences. Future research should incorporate patient and caregiver insights to refine the instrument.

Another limitation is the challenge of isolating the specific anxiogenic effects of polypharmacy. Participants were managing multiple chronic conditions of varying severity, making it difficult to determine whether their psychological distress stemmed from polypharmacy itself or underlying health issues. Future studies should consider more refined methods, such as longitudinal designs or more targeted analysis of polypharmacy as a distinct variable, to better assess its impact on anxiety levels in these populations.

While the PPDS is designed to measure distress specifically associated with polypharmacy rather than general anxiety, baseline anxiety levels could still serve as a confounding factor. Trait anxiety, as an individual’s predisposition to experience anxiety, may influence how patients perceive and report their distress, introducing variability into the findings. Therefore, individual differences in anxiety predisposition could impact the accuracy of the results. Future studies should incorporate trait anxiety assessments, such as the State-Trait Anxiety Inventory (STAI), to better account for these individual differences and control for the potential influence of trait anxiety on distress levels.

### 4.2. Implications for Research and Practice

The PPDS is a valuable tool for both clinical practice and research in the context of polypharmacy. In clinical settings, it can be used to assess the psychological impact of complex medication regimens, particularly among patients who may be more vulnerable to emotional distress due to the burden of multiple medications. By identifying individuals at risk, healthcare providers can implement personalized interventions, such as counseling, medication reviews, or shared decision making, to help alleviate distress and improve the patients’ overall well-being. The PPDS also enables the monitoring of psychological distress over time, offering a quantitative method to evaluate the effectiveness of interventions aimed at reducing polypharmacy-related distress. This allows clinicians to refine care strategies and ensure that patients receive appropriate and effective support. Furthermore, the PPDS can be incorporated into a broader standardized care protocol that integrates mental health considerations, especially in geriatric populations where the psychological impact of polypharmacy is often underrecognized. As a psychometrically sound measure, the PPDS captures patient-reported experiences, such as anxiety, worry, and frustration related to medication use, serving as a practical screening tool in both clinical and community settings. Its integration into routine care aligns with efforts to advance patient-centered approaches, particularly in chronic disease management (Fortin & Stewart, 2021), where understanding the emotional dimensions of treatment can significantly improve adherence and outcomes. By systematically addressing the subjective burden of polypharmacy, the PPDS contributes to more responsive, holistic, and person focused care.

From a research perspective, the PPDS may facilitate the exploration of relationships between polypharmacy-related psychological distress and various influencing factors, including individual characteristics, medication-related factors, and social or environmental determinants. Previous findings have linked negative emotional states with multiple medications (Van Wilder et al., 2022), and advances in measures, such as the current work, could support and provide research evidence. Thus, the PPDS can be used to explore the relationships between polypharmacy-related psychological distress and various factors, such as individual characteristics, medication-related factors, and social and environmental factors. By identifying the factors that contribute to psychological distress, researchers can gain a deeper understanding of the underlying causes and mechanisms, which is crucial for developing targeted interventions. Research findings can inform the development of evidence-based interventions designed to reduce psychological distress, tailored to address specific factors identified through research. Ultimately, the goal of research using the PPDS is to improve the overall well-being of individuals with polypharmacy by reducing psychological distress and enhancing their quality of life.

## 5. Conclusions

This study has introduced a brief instrument for evaluating and quantifying the psychological impact of polypharmacy. With further validation, this instrument holds the potential to not only improve the care and well-being of individuals dealing with polypharmacy but to deepen our understanding of the psychological effects of experiencing complex medication schedules. Further research is essential to validate its effectiveness across different populations and settings.

## Figures and Tables

**Table 1:** Polypharmacy-related Psychological Distress Scale (PPDS).

Please read each item carefully and respond based on your most genuine feelings over the past 14 days by marking a “√” in the corresponding box.						
1	Have you felt worried or nervous in the past two weeks due to taking a lot of medications every day?	□ Completely agree	□ Agree	□ Unclear	□ Disagree	□ Completely disagree
2	Have you felt worried or nervous in the past two weeks because of the names and usages of different medications?	□ Completely agree	□ Agree	□ Unclear	□ Disagree	□ Completely disagree
3	Have you felt worried or nervous in the past two weeks about taking medications on time and in the correct dosage?	□ Completely agree	□ Agree	□ Unclear	□ Disagree	□ Completely disagree
4	Have you felt worried or nervous in the past two weeks about the necessary precautions when taking medications?	□ Completely agree	□ Agree	□ Unclear	□ Disagree	□ Completely disagree

**Table 2:** Sociodemographic characteristics of the participants.

Variables		Number (n)	Percentage (%)
Age ranges (years)	60–69	22	24.2
	70–79	32	35.2
	80–89	31	34.0
	>90	6	6.6
Sex	Male	54	59.3
	Female	37	40.7
Living status	Alone	9	9.9
	With family	71	78.0
	Nursing home	11	12.1
Education level	Illiteracy	28	30.8
	Elementary school	37	40.7
	Junior high school	19	20.9
	Senior high school	7	7.6
Duration of chronic diseases (years)	<1	4	4.3
	1–5	19	20.9
	6–10	25	27.5
	>10	43	47.3
Multimorbidity *	Yes	81	89.0
	No	10	11.0

* Co-existence of two or more chronic diseases.

**Table 3:** Distribution of Responses Across Four PPDS Items.

Items	Completely Disagree (1)	Disagree (2)	Unsure (3)	Agree (4)	Completely Agree (5)
1. Have you felt worried or nervous in the past two weeks due to taking a lot of medications every day?	19 (20.9%)	15 (16.5%)	33 (36.3%)	22 (24.2%)	2 (2.2%)
2. Have you felt worried or nervous in the past two weeks because of the names and usages of different medications?	19 (20.9%)	13 (14.3%)	35 (38.5%)	22 (24.2%)	2 (2.2%)
3. Have you felt worried or nervous in the past two weeks about taking medications on time and in the correct dosage?	10 (11.0%)	11 (12.1%)	40 (44.0%)	24 (26.4%)	6 (6.6%)
4. Have you felt worried or nervous in the past two weeks about the necessary precautions when taking medications?	4 (4.4%)	8 (8.8%)	41 (45.1%)	23 (25.3%)	15 (16.5%)

**Table 4:** Results of internal consistency.

Items	Mean	SD	Corrected Item-Total	Alpha if Item Deleted	McDonald’s Omega
1. Have you felt worried or nervous in the past two weeks due to taking a lot of medications every day?	2.69	1.15	0.702	0.684	0.924
2. Have you felt worried or nervous in the past two weeks because of the names and usages of different medications?	2.65	1.13	0.692	0.691	0.909
3. Have you felt worried or nervous in the past two weeks about taking medications on time and in the correct dosage?	2.88	1.12	0.670	0.702	0.922
4. Have you felt worried or nervous in the past two weeks about the necessary precautions when taking medications?	3.20	1.11	0.359	0.849	0.920

**Table 5:** Results of inter-item correlation.

	PPDS Total	Item 1	Item 2	Item 3	Item 4
PPDS total	1	0.809 **	0.852 **	0.755 **	0.390 **
Item 1	0.809 **	1	0.938 **	0.383 **	−0.108
Item 2	0.852 **	0.938 **	1	0.441 **	−0.038
Item 3	0.755 **	0.383 **	0.441 **	1	0.325 **
Item 4	0.390 **	−0.108	−0.038	0.325 **	1

**. Correlation is significant at the 0.01 level (2-tailed).

## Data Availability

The data that support the findings of this study are available from the corresponding author, upon reasonable request.
